# Narrative Review Comparing Principles and Instruments Used in Three Active Surveillance and Control Programmes for Non-EU-regulated Diseases in the Danish Cattle Population

**DOI:** 10.3389/fvets.2021.685857

**Published:** 2021-07-19

**Authors:** Liza Rosenbaum Nielsen, Hans Houe, Søren Saxmose Nielsen

**Affiliations:** Section for Animal Welfare and Disease Control, Department of Veterinary and Animal Sciences, Faculty of Health and Medical Sciences, University of Copenhagen, Frederiksberg, Denmark

**Keywords:** disease control, non-EU-regulated, BVDV, paratuberculosis (MAP), *Salmonella* Dublin

## Abstract

The objective of this paper is to provide a comparative review of three active surveillance and control programmes in the Danish cattle sector to highlight important differences for decision makers to develop successful programmes. The focus is on differences in purpose, principles, design and instruments applied to achieve the goals stated for each programme for bovine viral diarrhoea (BVDV), paratuberculosis and *Salmonella* Dublin. The purposes of the programmes are to reduce economic consequences and improve animal welfare, and for *S*. Dublin also to prevent zoonotic risk, with varying importance as motivation for the programmes over time. The targets of the BVDV and *S*. Dublin programmes have been to eradicate the diseases from the Danish cattle population. This goal was successfully reached for BVDV in 2006 where the programme was changed to a surveillance programme after 12 years with an active control programme. The *S*. Dublin dairy herd-level prevalence decreased from 25% in 2003 to 6% in 2015, just before the milk quota system was abandoned. Over the last 5 years, the prevalence has increased to 8–9% test-positive dairy herds. It is mandatory to participate, and frequent updates of legislative orders were used over two decades as critical instruments in those two programmes. In contrast, participation in the paratuberculosis programme is voluntary and the goals are to promote participation and reduce the prevalence and economic and welfare consequences of the disease. The daily administration of all three programmes is carried out by the major farmers' organisation, who organise surveillance, IT-solutions and other control tools, projects and communication in collaboration with researchers from the universities, laboratories and, for BVDV and *S*. Dublin, the veterinary authorities. Differences among the programme designs and instruments are mainly due to the environmental component of paratuberculosis and *S*. Dublin, as the bacteria able to survive for extended periods outside the host. This extra diffuse source of infection increases the demand for persistent and daily hygiene and management efforts. The lower test sensitivities (than for BVDV) lead to a requirement to perform repeated testing of herds and animals over longer time periods calling for withstanding motivation among farmers.

## Introduction

Successful control and eradication of several infectious cattle diseases achieved in the past century in Denmark include many diseases, for example eradication of bovine tuberculosis (bTB), enzootic bovine leucosis (EBL), infectious bovine rhinotracheitis (IBR) and bovine brucellosis (1). Principles for effective disease control and eradication approaches in cattle have been described based on experiences from those programmes as well as experiences from ongoing control and eradication programmes (2). The principles include well-performing components integrated in the programmes, such as (i) motivated stakeholders and actors, (ii) efficient biosecurity measures based on knowledge of transmission mechanisms, patterns and risk factors for the disease in question, (iii) fit-for-purpose test-strategies, and (iv) resources to deal with logistic challenges such as collection of samples, handling of testing and test results as well as preparation of IT-systems for reporting of test results in a uniform and fit-for-farmer format. Furthermore, education and training of essential actors to acquire competences in practical and feasible disease control management have been important elements in the communication with stakeholders about the aim, target and effective measures taken in the programmes (2). Close collaboration between research institutes, authorities, laboratories and cattle sector institutions has contributed to developing, evaluating and adjusting these components to keep the programmes active and updated over extended periods and phases of the programmes. Although the overall principles for disease control and eradication are similar for different diseases, the actors and decision makers must understand the specific characteristics of each disease in sufficient detail to implement and carry through an effective control and eradication programme. Experiences from one successful eradication programme are not always directly transferable to or sufficient for another programme for different reasons that will be addressed below.

The objective of this review was to characterise three surveillance and control/eradication programmes that were active in the Danish cattle sector at the time of writing. The focus is on the comparison of the programmes in terms of purposes, targets, principles, design and instruments applied to achieve the goals stated for each programme for bovine viral diarrhoea (BVD), paratuberculosis and *Salmonella* (*S*.) Dublin. We chose these three diseases to represent a group of diseases not regulated by the EU and known to have been established in the Danish cattle population with a high or medium high occurrence and impact. The decision to initiate costly control and eradication programmes for these three diseases was not obvious without a comprehensive analysis of all relevant aspects as outlined. Some of these aspects became evident during the lifetime of the programmes, often before adjusting the programme instruments.

## Cattle Demographics in Denmark

**Figure 1:**
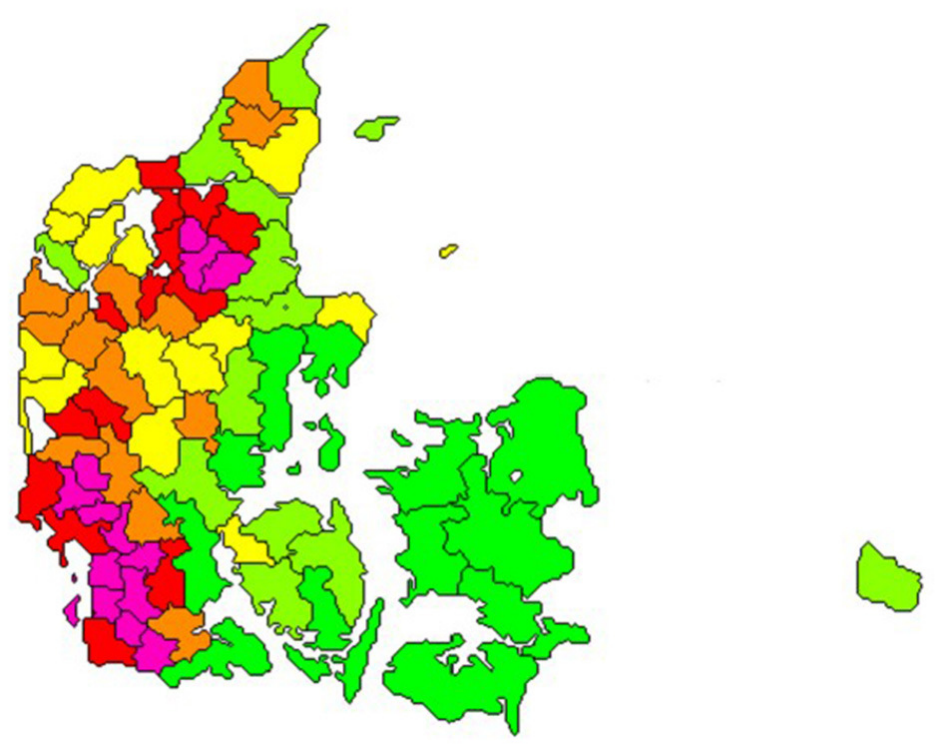
Number of cattle per square kilometre in all herd types in Denmark on the 23rd of February 2021. Bright green: areas with up to 19 cattle/km^2^, light green: >19–29, yellow: >29–44, orange >44–57, red: >57–86 and pink: areas with more than 86 cattle/km^2^. The size and shape of the geographical areas were generated to represent approximately equal number of cattle. (Source: SEGES, Aarhus, Denmark).

In 2020, the number of dairy cows was 565,000 and the total number of bovines in Denmark was 1,500,000 (3). These were mostly in 2,848 dairy herds, 994 dairy-heifer rearing properties, 9,438 beef herds, 619 veal calf herds, and 2,389 other herds (i.e., typically hobby herds). Furthermore, 133 cattle pasture premises were recorded (4). These registered pasture premises are typically shared by multiple herds. The clustered geographical distribution of cattle properties is illustrated in Figure 1.

## Disease Characteristics

The disease characteristics of relevance for control of infectious diseases have been outlined in a textbook describing and comparing aspects of the three diseases (2). In addition to the impact on animal welfare, farming profitability and food safety, the feasibility to establish biosecurity measures to mitigate spread of the pathogen, and test strategies to aid e.g., risk mitigation and surveillance must be addressed. One of the main considerations concerning biosecurity measures is whether the pathogen mostly spreads via live animals or whether it also survives in and spreads indirectly *via* the environment. The test performance should be evaluated both at the individual animal level and the herd level. Evaluation of the performance of the testing programme is also important at national or sector level in mandatory programmes. To ease comparison, characteristics of the agents and diseases of importance for controlling them are listed in Table 1, and key aspects and progress of the Danish programmes for the three diseases are summarised in Table 2.

**Table 1:** Comparative summary with non-exhaustive information about important characteristics of three infectious diseases under surveillance and/or control in Danish cattle during up until June 30, 2021.

**Important pathogen and disease characteristics**	**BVDV**	**Paratuberculosis**	***Salmonella* Dublin**
Pathogen	Single-stranded RNA virus belonging to genus Pesitivirus under family *Flaviviridae*.	Intracellular, acid-fast bacterium.Slow growth on solid media (8–16 weeks); slightly faster on liquid media (>5 weeks).	Intracellular, Gramme-negative bacterium, grows in wet/humid, warm conditions with organic materials present.
Pathogenesis	Transient infection via oro-nasal route or transplacental infection causing persistent infection.	Primarily faecal-oral transmission, but also vertical transmission *in utero*. Incubation period typically 2–5 years.	Faecal-oral transmission, short incubation time (1–2 days), can generate latent or persistently infected carriers.
Host susceptibility and clinical signs	Hosts: cattle and other domestic and wild ungulates. Several clinical manifestations incl. fever, salivation, diarrhoea, abortions, congenital defects, unthriftiness, mucosal lesions and death.	Ruminants primarily affected, with calves more susceptible than adults. Clinical signs are predominantly intermittent diarrhoea with loss of weight moving towards persistent diarrhoea, emaciation and death.	Host-adapted to cattle, calves more susceptible than adult, all ages can be infected – some get acutely or chronically ill (mainly with diarrhoea, fever, pneumonia, arthritis, distal skin necrosis, septicaemia).
Environmental survival	From days to few weeks, e.g., in slurry.	More than 200 days under moist conditions such as in slurry and manure.	Yearlong survival in manure. Proliferates at pH 5–6 in milk, inhibited at lower pH.
Main risk factors	Movement of cattle, and to some extent indirect transmission.	Movement of cattle; cows' faecal contamination of the calves environment; use of milk and colostrum from infected cows.	Movement/purchase of cattle, high animal density, poor hygiene, low immunity in calves.
Available tests	ELISA tests and PCR.	Indirect ELISA (bacteriological culture and PCR, but not in Denmark).	Indirect ELISA (serum, bulk tank and individual cows' milk. Bacteriological culture and PCR.
Main motivations to control in Denmark	Economic losses, severity of disease, initial high prevalence, later also animal welfare.	Production losses, end-stage severity of disease (animal welfare), potential food safety issue.	Food safety, initial high prevalence, severity of disease (animal welfare), and later in programme economic losses also a motivation.

**Table 2:** Overview for comparison of key features and progress of control programmes for BVDV, paratuberculosis and *Salmonella* Dublin in Danish dairy cattle farms up until June 30, 2021 (i.e., the information is non-exhaustive).

**Key features of the disease control programmes**	**BVDV**	**Paratuberculosis**	***Salmonella* Dublin**
Main biosecurity measures	Avoid contact with cattle from other farms. Hygiene of instruments and other equipment used in different farms.	Reduce purchase of livestock; avoid cows' faecal contamination of calves' environment, especially at calving. Avoid use of milk and colostrum from test-positive cows.Cull repeat-positive.	Stop purchase from test-positive farms, rigorous hygiene and sectioning of animals in management groups to lower/stop transmission, good calf and calving management and hygiene. Pasteurisation of milk used in some farms. Culling of suspected carriers in some herds.
Test-strategies	Step wise testing of bulk tank milk, spot sampling of young stock and testing of individual animals.	Repeated testing using indirect ELISA on individual cows' milk from the milk recording system.Repeatedly test-positive cows culled if possible; all test-positives considered potentially infectious and measures to reduce transmission from these are pivotal.	Bulk-tank milk antibody tests every 3 months in all dairy herds, blood sampling at abattoirs or on-farm in non-dairy herds. Testing calves negative required before test-negative status of herd can be obtained. Bacteriological culture mainly used for herds with high risk or clinical suspicions, “salmonellosis.” In some herds, repeated testing used for detection of suspected carriers.
Mandatory/voluntary	Mandatory surveillance and control programme of all cattle herds. Legislation in place from early on and updated regularly.	Voluntary surveillance and control programme.	Mandatory surveillance and control of all cattle herd. Legislation in place from early on and updated regularly to target and strengthen control measures.
Feasibility	Requires focus on clarification of herd infection status and control of cattle movements.	Requires persistent focus on hygiene; testing can be used to identify high-risk animals to make the efforts risk-based. Uncertainty in test interpretation must be accepted.	Requires daily, persistent focus on hygiene, reduced animal contacts and follow-up for years. Challenging in large, multi-site farms with many animal movements. Some uncertainty in test interpretation must be accepted.
Prevalence/progress of programme	Since 2006: Zero or few sporadic cases per year after successful control programme.	June 2021: 60–70% of herds deemed infected; mean within-herd prevalences in herds in control programme <5%.	At surveillance initiation in 2002: 25% test-positive dairy herds. June 2021: 9% test-positive dairy herds.

### BVDV

The causative agent belongs to the genus pestivirus belonging to the family flaviviridae. Among the 11 species of pestivirus, three are known to infect primarily cattle, namely pestivirus A (former BVDV-1), pestivirus B (former BVDV-2) and pestivirus H (Hobi-like pestivirus). Each of these three species can be sub-divided into several subtypes (5, 6).

The main characteristics of BVDV infections are the existence of two types of infection courses, namely transient infection and persistent infection (PI). Transient infection occurs after infection of immunocompetent animals. Shortly after infection, the animal becomes viraemic, usually for 2–3 weeks, e.g., (7). Some animals may develop diarrhoea after an incubation period of few days. This phase is followed by a rise in antibody levels over the next many weeks (8). The antibodies are long lasting and often the animal will be antibody positive for the rest of its life (9).

If a foetus becomes infected between typically day 25–90 and occasionally up to day 125 of foetal life (i.e., before development of immunocompetence), it will become immunotolerant for the rest of its life (both pre- and post-natally) (10, 11). The animal therefore becomes persistently infected and will, except for a few months after colostrum uptake, express life-long viraemia and will excrete the virus through the airways and body fluids (12–14). The PI animals can show a variety of clinical signs including growth retardation, ill thrift and increased susceptibility to other infections. If the initial infection is later followed by infection with a so-called cytopathogenic type of the virus, the animals will often develop the fatal condition mucosal disease (15, 16).

BVDV survives for a relatively short time in the environment, e.g., one study showed from days to very few weeks (17).

### Paratuberculosis

Paratuberculosis is a chronic infection in cattle and other ruminants, caused by *Mycobacterium avium* subsp. *paratuberculosis* (MAP) (18). The disease is characterised by an incubation period of usually at least 2 years, but 2.5–4.5 years is the age-range where infections are more likely to be detected via faecal shedding and sero-responses (19). Reduction in milk yield, which has been estimated to an average of 6% in infected animals (20), a reduction in slaughter weight and value (21), and an inconclusive zoonotic potential (22) has made this disease a target of disease control programmes globally (23). Apart from these effects, MAP can have a severe impact on animal welfare, as clinical disease characterised by weight loss, poor body condition, chronic wasting, and intermittent diarrhoea, followed by emaciation and pipe stream diarrhoea eventually leading to death occur in a proportion of infected animals (24).

The faecal-oral route is the primary route of infection, and calves <3–6 months old are considered more susceptible to infection compared to older herd-mates (18), although adults may also be susceptible to high doses of MAP resulting in infection (25). Transfer of MAP by mechanisms such as pinocytosis in the first 24 h of life has for example been suggested (18). MAP is primarily excreted in faeces of adult cattle, although excretion in colostrum and milk also frequently occurs (26, 27), whereby susceptible calves can be infected. Furthermore, transmission can occur *in utero*, even from cows not demonstrating clinical signs of MAP infection (26, 28). MAP can survive for at least 55 weeks in fully dry and shady environment, and has been demonstrated to survive 9 weeks on grass in 70% shade (29).

### *Salmonella* Dublin

*S*. Dublin is the most commonly detected salmonella serovar in Danish cattle. It is host-adapted to cattle, but sometimes causes salmonellosis in other species including humans, mink, sheep and wildlife (30–33). The course of the infection is age and dose dependant and varies between infected individuals (34–36). The incubation period is 1–5 days depending on the dose, infection route, prior infection and individual variation (37, 38). The time from uptake until faecal shedding of bacteria begins is 1–7 days (37), which means that salmonella can spread rapidly within and between herds.

Infected animals can experience different infection and disease progression stages: short-term (1–3 weeks) infection, which can be asymptomatic (subclinical) or acute disease characterised by mild or intermittent clinical signs that the animal may recover from with no or supportive treatment. Short-term infections can also be peracute or acute with severe disease, which is difficult to treat due to septicaemia and invasive infection and associated with high case-mortality (39). Less commonly, chronic clinical infection lasting weeks to months with persistent clinical signs of varying severity is observed (40). These animals are often euthanised, as prognosis is poor. A low percentage of infected cattle become persistent carriers of the pathogen for months to years. These may excrete bacteria intermittently (often referred to as “latent carriers”) or in rare cases more or less continuously (often referred to as “active carriers”) (34, 41, 42). Cattle with asymptomatic infection or acute mild disease shed the bacteria for on average 17 days (43). The active and latent carriers do not necessarily exhibit clinical signs, but may have been ill previously. Most likely salmonellosis predisposes to development of the carrier stages (34, 37, 38).

Environmental spread is also important to consider. *S*. Dublin can survive for 1–12 weeks (depending on the weather conditions) in grass and soil after being spread with slurry onto pastures, and animals grassing such contaminated pastures can become infected and shed the bacteria themselves (44). *S*. Dublin has also been shown to survive for years in dried faecal material in the barn environment (45).

### Comparative Considerations

The three diseases vary considerably with regard to the causal pathogen, pathogenesis, incubation periods, duration of infection and clinical manifestations, transmission patterns and environmental survival of the pathogens. BVDV and *S*. Dublin share some features in terms of acuteness of disease and rapid spread between animals and herds of animals, which make them easier to diagnose based on clinical suspicion sooner after the animals/herds become infected than paratuberculosis.

MAP and *S*. Dublin, on the other hand, share environmental spread mechanisms, due to the ability of the pathogens to survive for extended time-periods in the surroundings of the host. These two pathogens also share the primary infection route in that the faecal-oral route is the most common way for susceptible animals to become infected. *In utero* infection can occur for all three diseases. However, it is a most prominent feature of importance for the control of BVDV, which is unique in having a well-defined chronic stage in the form of immunotolerance and persistent infection. On the one hand side it is a strength for spreading the infection, but it also showed to be an easy target for intervention.

## Motivation Among Stakeholders – Realisation of the Clinical, Economical and Food Safety Importance

### Motivations to Control BVDV

In Denmark, the economic losses were the main driver for starting the BVDV control programs. As the first control programme was launched already in the beginning of the 1990'ies, the data on motivation dates many years back. Many BVDV infections are subclinical. Therefore, the actual impact of the virus was initially difficult to comprehend. In order to obtain a more accurate picture of the occurrence of infection, screening of dairy herds with unknown infection status was carried out in 1988 (46). That study showed that more than 50% of herds had PI animals, and all herds in the screening had antibody positive cows. Among individual animals, 1.4% of all cattle were identified as PI and more than 60% of individual animals were antibody positive. The study was relatively small including only 19 herds, but the epidemiological features were similar to later findings in Denmark and many other countries (47).

The impact on farming profitability was the primary driver for establishment of the programme in 1994. The financial consequences at national level have been calculated based on epidemiological studies and knowledge of the clinical and production effects. The annual national losses among dairy cattle in Denmark were estimated to be 13 million GBP per million calvings (48), while later reviews estimated the losses per cow in endemically infected herds as 30–60 EUR (49) and 10–40 USD per calving (50).

### Motivations to Control Paratuberculosis

In the end of the 1990'ies, many dairy farmers in Denmark did not want to officially recognise if their herd was infected with MAP. Pursuing the diagnosis was often avoided, because of fear of stigmatisation among peers and potential trade issues. MAP was not notifiable, except according to the act on purchase of goods, according to which all flaws associated with a sale of a good is notifiable. However, following the reporting of a high prevalence in 1998 (51, 52) and an even higher between-herd prevalence of 85% in 1999 (Nielsen et al., unpublished data), a general recognition of these high prevalences started to prevail. When the Danish programme was launched in 2006, 10% of the herds were initially enrolled, but this number increased to almost 30% (including 35% of cows) before 2010 (53), suggesting that the fear of the stigmatisation associated to the disease had diminished. The majority of 1,177 farmers reporting why they participated in the programme said they did so to (multiple responses possible): (1) increase animal health (91%), (2) be certified free of MAP infection within 4–10 years (87%); and (3) avoid production losses (86%) (54). Apart from these challenges, MAP infections may interfere with tuberculin testing for bovine tuberculosis (55). However, Denmark has been recognised officially free from bovine tuberculosis since 1980 (1), and therefore, this is not a concern.

### Motivations to Control *Salmonella* Dublin

Before the Danish *S*. Dublin surveillance programme was initiated in October 2002, there were increasing concerns about morbidity, mortality and persisting infections in test-positive farms as well as research demonstrating more than 20% of the dairy herds being test-positive to the disease (56). The consequences of the disease vary a lot between individuals and between affected herds (57). Abortion is common when infection occurs in pregnant heifers or cows, and can occur at any time during the pregnancy (34, 35). The bacterium has been known to be a severe zoonosis with a high case fatality for many years (58, 59). This aspect, however, became clearer in Denmark after the programme was initiated (32, 60), which underpinned the decisions to change the strategy from surveillance to a control programme. The sources of infection for humans are contaminated beef or unpasteurised milk products (59) or direct contact with infected cattle (61). The annual number of recorded human cases in Denmark varied between 19 and 50 from 2001 to 2020 (62). More than 90% of the Danish human cases are attributed to domestically produced beef, the rest are thought to be travel-related (63).

Research carried out in 2009–2011 demonstrated larger production losses and hence higher economic effects of *S*. Dublin than hitherto anticipated in test-positive dairy herds (64). Lactating cows might experience a significant drop in milk yield in most of the infection stages described above probably even when clinical signs are not apparent, in dairy herds with clear indications of *S*. Dublin introduction to the herd based on surveillance test results. Simulation modelling demonstrated marked gross margin losses upon introduction of *S*. Dublin to dairy herds, often for years after the infection was introduced (65). However, the production losses may not be noticed by the farmer due to the delayed effects of calf disease on milk production and fertility in dairy herds and the protracted course of the infection in many herds.

The combined issues with food safety and production losses and a need to be able to better control the spread of *S*. Dublin between cattle farms were the drivers of decisions to strengthen the surveillance into an active and mandatory control programme aiming for eventual eradication of the disease from the Danish cattle population. At the time of writing there were ~9% test-positive dairy herds in the country. There is also a working group and a steering group working under leadership of the Veterinary and Food Administration to improve the control efforts to protect non-infected herds from becoming infected and to encourage farmers in infected herds to make the needed efforts to stop the spread of the infection within and out of their farms.

### Comparative Considerations

The weight of importance of the three main drivers (farming profitability, animal welfare and zoonotic potential) vary considerably between the three diseases. For BVDV, is was at originally solely the very clear effects on production, which were made obvious from both a number of case stories with severe outbreaks, and also calculation of economic losses showing that intervention would be cost efficient. Animal welfare was not a big issue when the first BVDV campaigns were initiated, but they it would certainly have been today. For paratuberculosis and *S*. Dublin, it may to a greater extent have been a combination of the three drivers, both having a medium impact on production and animal welfare, while the zoonotic potential was a particular driver for *S*. Dublin, but also a potential but unspoken of concern for paratuberculosis. Furthermore, the impact of BVDV infections can rapidly become clear in the individual herd, and so can the effect of control measures taken. For *S*. Dublin and paratuberculosis, the effects of introduction of *S*. Dublin or MAP may not be so obvious, and only years after the pathogens have spread to a larger part of the herd, actions are undertaken, unless surveillance and mandatory actions are in place.

Vaccination has never been used for BVDV, because a vaccination study conducted in 1992 resulted in the production of many PI calves (66). Vaccination for MAP was discontinued in 2008 due to the interference with the serological tests (67) and with tuberculin testing in case of export of animals (68). Vaccination against salmonella is not used for any food-producing animals in Denmark.

## Biosecurity Measures

### Breaking the Transmission Routes of BVDV

As stated earlier, BVDV only survives short time in the environment, i.e., from days to very few weeks (17). Therefore, transmission of BVDV is primarily via direct contact between susceptible animals and acutely infected or PI animals. PI animals are the most infectious source of BVDV in transmission (46, 69, 70). Other minor routes of transmission includes semen, embryos (12, 71) and short-distance airborne transmission (72). Whereas, some other ruminants, wildlife animals and pigs may be infected, they were not deemed to play a major role in the Danish control programme, because their infectious capacity was limited (66, 73, 74). A range of other sources of transmission are possible, e.g., indirect transmission by use of equipment, contaminated needles, medicine bottles and vaccines have been demonstrated to contribute to spread of BVDV (2).

Important biosecurity tools in the control program was that the disease was notifiable, and emphasis was on securing health certificates for animals before their movement to other herds or common pastures. Furthermore, focus was on keeping PI animals from pastures. Further, owners of infected herds should inform neighbours and visitors about their infection status. Purchased animals should be placed in quarantine in case that have been recently infected and purchased pregnant cattle must calve in isolation until the calf has been tested negative for BVDV.

For countries or areas where biosecurity measures are not considered sufficient to avoid spread of infection, a hybrid control program combining initial use of vaccines with other control elements has been suggested (75).

### Breaking the Transmission Routes of MAP

Between-herd transmission is primarily a result of movement of MAP infected livestock, and pre-movement testing may not be effective, because many infected animals have yet to have analytes detectable. For example, the diagnostic sensitivity of antibody-ELISA can be <5% in cattle <2 years of age (76), and these are often the animals that are purchased in dairy herds. Therefore, the primary instrument to control between-herd spread of MAP is via movement control. Because of generally high between-herd prevalences (77), and because of low diagnostic sensitivity for detection of infected animals (77), herd-specific freedom from infection can be difficult to ascertain in small herds or if testing is not done frequently (78). Therefore, a tool to reduce the risk of between-herd transmission would include frequent testing of the within-herd prevalence, and if a closed herd cannot be achieved, farmers should purchase livestock from low-prevalence herds and they may thereby be able to reduce the risk to levels, where infection can be cost-effectively controlled (79).

Mitigating within-herd transmission focuses primarily on reducing the risk of spread of manure from adult cattle to the more susceptible calves and/or young stock. While contact between calves and adults primarily occur in the calving area, removal of the calf as quickly as possible following birth can be required. Furthermore, the calf should be born in a clean calving pen, and the calf should also subsequently be protected from manure of the adults, e.g., housing of the calves should be in other facilities than those of the adult (80, 81). Additionally, calves should not be fed colostrum and milk of infectious dams, and infectious dams may also transmit MAP to their offspring (23). However, calves are still required to have colostrum. Their welfare increases if they can stay with their dam, and milk can be an inexpensive nutritious feed in early life. Yet, the only way to identify infectious adults are via testing. Therefore, risk mitigation can be done via a risk-based approach, where the listed practises are done only for test-positive cattle, and culling of a subset of these only is done to reduce spread of MAP while still retaining those that are less likely to excrete MAP (82, 83).

Specifically in Denmark, risk-based control in herds in the Danish control programme is done by testing cattle prior to dry-off (and calving) to have updated test results. All test-positive cattle should then: calve in a calving pen separated from other calving pens, have their calf removed immediately, not provide colostrum and milk to their offspring, be culled if they are repeated positive. Testing is done using a milk antibody ELISA, which has a high sensitivity of detection of infectious cattle (83). Furthermore, it is encouraged not to purchase livestock, but if livestock is purchased, it should be from tested and low-prevalence herds.

### Breaking the Transmission Routes of *S*. Dublin

The spread mechanisms of *S*. Dublin resemble those of MAP. Both pathogens spread mainly via manure. Therefore, movement of cattle, manure and manure-contaminated vehicles is the biggest risk factor for spread of this infectious agent. Thus, it makes sense that there is clear evidence of local spread of *S*. Dublin around test-positive herds (84), as well as spread between herds with linked trade/movement networks (85, 86). However, the exact source and time of the agent spread is usually difficult to pinpoint. Hence, the risk mitigation measures need to be comprehensive and include considerations of the environmental survival to have sufficient effect. Animals from infected (or test-positive) herds should not be allowed to be moved to other herds, shows, markets, pastures etc., where they can get in contact with susceptible animals or their manure can lead to indirect spread of bacteria.

Newborn and milk-fed calves are also the most susceptible to the infection, although all ages can become infected and spread the infection. Hence, control measures should always include continuous focus on potential ways that the newborn and young calves might become infected in the herd, when trying to control the infection (87). Therefore, the calving environment and young calf housing and management are weighted high in the risk assessment tool used most frequently in Danish farms (88). Heat treatment of colostrum and pasteurisation of milk may be helpful in some farms, where contamination is difficult to control (89).

Finally, it should be kept in mind that *S*. Dublin can cause severe invasive infections in humans. Farmers and others moving into an infected farm should be (made) aware of this potential risk and take necessary precautions, such as wearing gloves, washing hands and preventing inhalation of potentially contaminated aerosols (e.g., during high-pressure washing). Drinking unpasteurised milk from infected farms is an important risk to be aware of, as outbreaks of disease in humans have occurred through this source (59, 61).

### Comparative Biosecurity Considerations

Direct transmission between animals likely occurs easier for BVDV compared to *S*. Dublin and MAP. However, BVDV survives only shortly (days) outside the host, whereas both *S*. Dublin and MAP can survive for months up to years in the environment. The environmental survival and the structural changes (bigger and more multi-site farm structures) of the Danish cattle herds are plausible reasons for the difficulties in further reducing the *S*. Dublin prevalence in spite of the strict cattle movement-restricting control programme. Furthermore, the transmission routes differ greatly between MAP plus *S*. Dublin with the faecal-oral route being predominant and calves being more susceptible vs. BVDV with the pregnant dam playing a key role, if she becomes infected and produces a PI-calf, which can then maintain the infection in the herd if the PI-animal is not identified and removed. These differences are important to consider when prioritising biosecurity measures. Common to all three infections is the identification and removal of the most infectious animals, although this poses challenges in persistently *S*. Dublin-infected herds.

A closed herd policy towards BVDV and *S*. Dublin infected herds is strictly required to keep the infection out of naïve herds, and this is feasible with the reasonably accurate herd classification of test-negative herds that can serve as source herds for purchase of replacement animals (see next section). However, for paratuberculosis, the recommendation is to only purchase cattle from tested low-prevalence herds, because these herds pose a lower risk than non-tested and high-prevalence herds (79). An opportunity for establishing biosecurity for BVDV is the possibility of issuing test certificates for non-pregnant animals that in combination with a relative short quarantine can make purchase of animals possible with low risk of introduction of infection.

## Test Strategies

### Test Strategies for BVDV

A stepwise test strategy consisting of (1) antibody detection in bulk-tank milk (BTM), (2) spot test sampling of young stock, and (3) follow-up testing of individual animals proved highly efficient for classification of herd status as well as monitoring of free herds. However, to understand the test strategy, it is necessary to look at the test performance at animal level.

#### Testing at Animal Level

For BVDV infections, there are several diagnostic tests for detection of either virus or antibodies. Different ELISA's have been used both for detection of antigen and antibodies in the BVDV control and eradication programmes, because these techniques are relatively fast and inexpensive. Often these tests have high sensitivity and specificity. For example, the Danish antigen ELISA used initially showed a sensitivity and a specificity of 97.9 and 99.7 for detection of antigen when compared to virus isolation test, while the antibody blocking ELISA showed a sensitivity and a specificity of 96.5 and 97.5 when compared to serum neutralisation test for use in cattle (90). However, there may be exceptions in which the test is not accurate, for example in calves with presence of colostral antibodies. Antibody positive results from these animals may reflect either a transient infection, or colostral antibodies that may even prevent the detection of viral antigen. Therefore, interpretation in calves should be done with caution or repeated testing should be done (91).

One of the first assessments of the feasibility to use antibody ELISA was in the Samsø-project, where the cattle population of a small Danish island, in total 2,200 cattle, were tested. An almost perfect bimodal distribution of the antibody reaction was observed, which eased the use of the blocking ELISA to identify antibody positive and antibody negative cattle (66).

Later, testing in control programmes has increasingly been supplemented or replaced by rt-PCR tests, which have the advantage of higher analytical sensitivity (92).

#### Herd Level Strategy

Overall, the main objective is to determine if PI animals are present in a herd or not. The following tests have been used in a stepwise procedure in order to keep the cost of testing as low as possible:

- Detection of antibodies in BTM,
- Detection of antibodies in a spot sample of individual samples from young stock,
- Follow-up on individual animals.

For non-dairy herds, the testing starts with step b.

- Herd level diagnosis using antibody in BTM to detect herds with PI animals

Several studies have revealed a herd sensitivity (HSe) in the level of 0.8–0.9 for the detection of herds with PI animals. False negative test results can occur in herds with very young PI animals that have yet to transmit the BVD virus to other animals in the herd. However, if the BTM testing is repeated a few month later, it will be positive. On the contrary, the herd specificity (HSp) will often be low (even below 0.5). This is because herds will still have many antibody positive cows for 1–2 years after removal of the last PI animal and thus appear as false positive.

Therefore, the strategy of using BTM is that test-negative herds are repeatedly tested a few months later with a BTM test to reveal false negative herds. If still negative, they can be declared non-infected and be transferred to monitoring. Herds that are BTM positive should have follow up testing using the young stock test as described in next section.

(b) Herd level diagnosis based on testing antibodies in individual samples from young stock

Testing a proportion of young animals (after the antibody colostral period) for the presence of antibodies to indirectly indicate presence or absence of PI animals in the herd is often referred to as “spot testing.” The HSe will be high and even higher than BTM testing, because the PI calves are very efficient in transmitting the infection to other calves, i.e., there are few false negatives. But the HSp will also be relatively high, because the young animals must have seroconverted recently. When there are no PI animals in the herd, antibody negative young stock will appear as soon as they have lost their colostral antibodies. For the young stock spot test, HSe and HSp of 0.93 and 1, respectively, have been reported (93).

Therefore, if the young stock test is negative, the herd continues with monitoring and if the young stock test is positive, a follow up of individual animal testing should be done.

(c) Follow up testing to identify virus positive animals

When a herd is suspected of harbouring PI animals, testing of individual animals is necessary. Different testing strategies can be pursued. As colostral antibodies can hinder virus detection using antigen ELISA up until 8 months of age, these animals must either be tested later or a PCR test can be used, as it is not affected by the presence of colostral antibodies. In animals older than 8 months, virus detection in PI animals can occur with very high accuracy. Also, calves born until 9 months after the removal of the last PI animal should be tested as early as possible, preferably by PCR to avoid colostral antibodies hindering virus detection.

The methods of continuous monitoring used to confirm infection-free status follow the same principles as those used to establish initial herd status. Based on the testing objectives described in the previous section, a flow diagram for the decisions under (a), (b) and (c) was set up, see Figure 11.2.2 p. 125 in (2).

The current surveillance scheme requires testing of every dairy herd for BVDV antibodies in BTM samples 4 times per year. This is done through collection of milk quality samples during December, March, June and September. The surveillance of non-dairy herds is done through analysis of blood samples collected at the slaughterhouses when cattle are sent to slaughter (94).

### Test Strategies for Paratuberculosis

There are two primary purposes with testing in the Danish control programme for paratuberculosis:

1. early detection of infectious animals; and
2. classification of herds as low-prevalence herds that can serve as sources of low-risk animals for purchase of replacement livestock.

To achieve the former, frequent testing was used in the first 14 years of the programme, using an in-expensive test (milk ELISA, price ~3.75 EUR/test including sampling). To achieve the latter, whole-herd milk ELISA testing or testing of at least 150 animals per year to classify a herd based on the prevalence. Agent-detecting tests such as culture and PCR were not considered, because of the test costs being almost 10 times the costs of ELISA including sampling, see (78). Instead, “confirmation” of testing was based on the repeated testing scheme, and major efforts were made to explain the risk of false-positives. This was, for example, done via standardised laboratory reports developed to assist directly in management on-farm (95).

**Figure 2:**
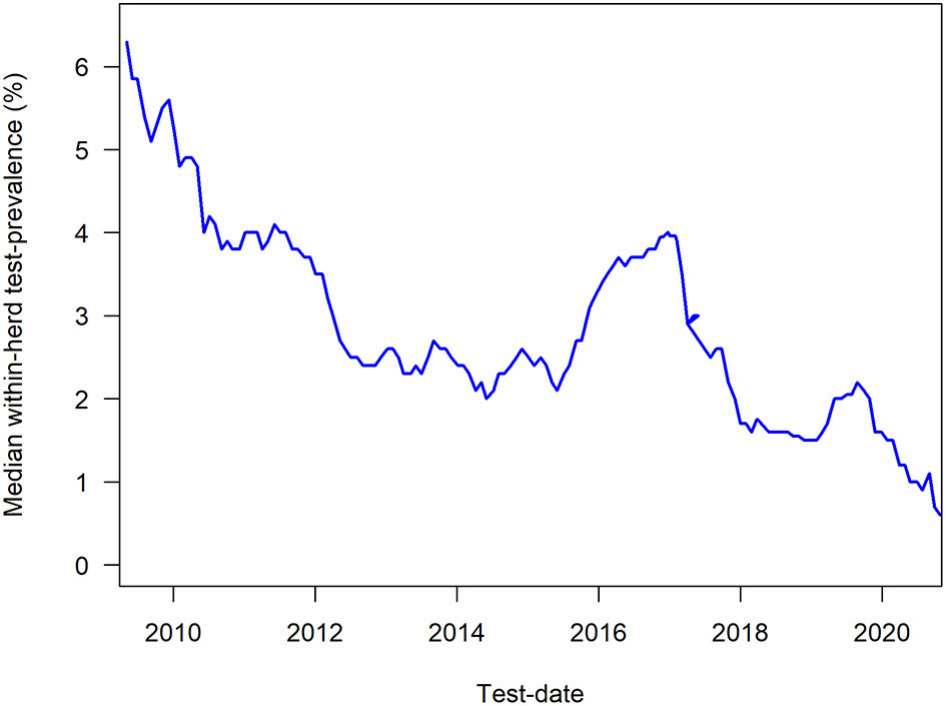
Median within-herd test-prevalence of antibody ELISA test results from June 2009 to February 2021 among herds in the Danish control programme on paratuberculosis.

The milk ELISA test used is ID-Screen^®^ Paratuberculosis Indirect (ID-Vet, Grabbels, France), which has been estimated to on average have a mean effective sensitivity to detect infected cows of 0.60 (96) based on the age-distribution in the lactating population of cows. The milk ELISA has an age-dependent sensitivity from 0.33 at 2 years of age, increasing to 0.94 at 5 years of age, relative to the cows that are deemed to ever develop antibodies; the associated specificity has been estimated to 0.9866 (76). The age-specific sensitivity and specificity can be used to calculate the probability that are herd is free of MAP infection, using the approach described in elsewhere (78), and this probability is reported to the farmers along with the calculated true prevalence (apparent prevalence corrected for sensitivity and specificity). From 2020, the Danish programme was updated to primarily recommend that cattle are tested prior to dry-off, so they have an updated test-result when they are calving, to enable risk-based management. The cows are automatically identified based on their stage in gestation, as listed in the Danish Cattle Database. Herds cannot be deemed “free of MAP infection,” although the true prevalence can be estimated to 0%, and the probability of freedom can be very high. The development in the within-herd test-prevalence among herds in the programme is shown in Figure 2.

### Test Strategies for *Salmonella* Dublin

Overall, there are four purposes for using diagnostic tests for *S*. Dublin in surveillance and eradication programmes as described below.

#### Surveillance of Herds

The Danish *S*. Dublin herd classification programme is based on antibody measurements using a *Salmonella enterica* subsp. *enterica* serogroup-D in-house ELISA (Eurofins, Vejen, Denmark), because the bacteriological detection methods are more costly and have lower sensitivity (97, 98). Although cross-reactions with other serotypes can occur, *S*. Dublin is by far the most commonly detected serotype of *Salmonella* strains detected in Danish cattle farms, and hence the programme is still considered to mainly target *S*. Dublin. Repeated measurements over time are used, because documentation and research projects have shown that it is not sufficiently accurate to base the herd classification on a single BTM sample or a single cross-sectional sample of calves (97–99). Dairy herds are BTM tested four times per year, and are placed in “Level 1” (test-negative) if the average of the last four BTM ELISA results is below 25 ODC% *and* the latest sample does not have an ODC% value that is more than 20 above the average of the previous three BTMs. This latter is sometimes referred to as “the jump criteria” (98) and it gives higher weight to the most recent measurement to enable easier detection of new herd infections. Herds that do not live up to the Level 1-criteria are placed in “Level 2.” The *S*. Dublin dairy herd-level prevalence decreased from a high of 25% in 2003 to as low as 6% test-positive cattle properties in 2015, before the milk quota system was abandoned. Since then the prevalence increased to 9% test-positive dairy herds by March 2021.

**Figure 3:**
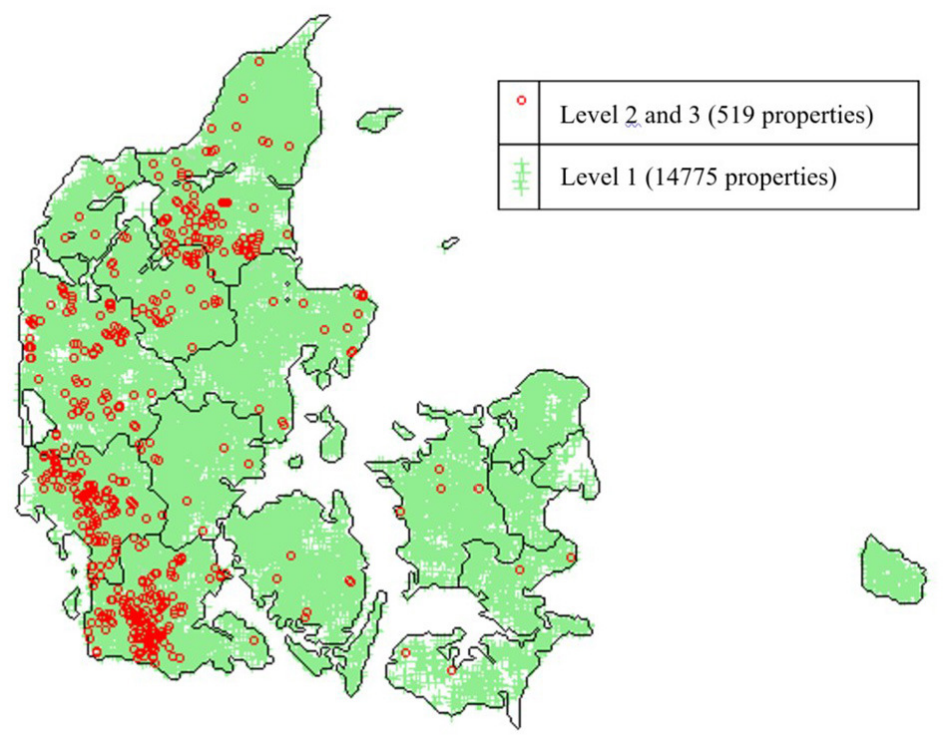
Map of the distribution of *S*. Dublin surveillance levels on properties with cattle in Denmark on the 24th of March 2021. Green marks: *S*. Dublin Level 1, Red rings: Level 2 or 3 (test-positive and other properties not in Level 1). (Source: SEGES, Aarhus, Denmark).

Non-dairy herds are classified according to test-results from antibody measurements of blood samples collected from slaughtered animals according to automatically selection generated by an IT-system linked to the Danish Cattle Database. Blood samples used for BVDV testing are partly used for the *S*. Dublin programme. The IT-system informs the laboratory about which samples should be tested for which diseases. All tested blood samples must be below the cut-off value of 50 ODC%, which at animal level gives a sensitivity of around 0.75–0.77 and a specificity of ~0.95–0.99 depending on the age of the animal (100, 101). Figure 3 illustrates the distribution of test-positive and test-negative cattle herds in Denmark.

#### Identification of Animal Management Groups With Ongoing Transmission

Test-strategies used within infected herds to support control of the infection should ideally be herd-specific, as within-prevalence varies a lot between age groups and over time, and are highly dependent on herd structures, logistics, group sizes, separation of groups of animals and hygiene (102, 103). ELISA-testing for antibodies directed against *S*. Dublin in serum and milk samples is used frequently both in the surveillance programme as described above, and as part of on-farm control strategies, as it is more sensitive than faecal culture methods to detect recent exposure to the bacteria (101). In general, calves between the age of 3–6 months should be tested regularly until there is sufficient evidence that the calves are no longer becoming infected between birth and 3 months of age. Once this is established, it is possible to start testing older age groups to see, if there are other groups of animals in which the infection is still spreading.

It is not useful to test calves younger than 3 months with antibody tests, because they are rarely able to produce measurable antibody responses against *S*. Dublin until around 11–12 weeks of age (104). The sample size ideally should be calculated to fit the size of the herd or group of animals to be tested, as both the sensitivity and specificity of the ELISA need to be considered in the interpretation of the test-results (99, 100). In some farms, it makes sense to test heifers before and after calving to investigate whether infection happens in the calving area. It may also be useful to test the cows with individual milk-ELISA to evaluate the infection pressure in the cow barn and to identify possible patterns in the antibody measurements.

#### Evaluation of Effect of Control Measures

It is specified in the legislation, which herds and how often the herds need to test young stock to document effect of control measures. In the new legislation expected to be implemented in July 2021, Level 2 herds will have to test calves every 3 months until they have been test-negative in two consecutive test-intervals and then young stock above 6 months old every 6 months until they are also test-negative. Only herds with the BTM test scheme and the young stock all living up to the criteria will be place in Level 1. Previously, a sample size of eight blood samples has been used for groups of calves bigger than 10 animals. However, in the updated legislation the sample sizes will be bigger for bigger groups of animals to avoid missing infection exposures, when prevalence is low.

#### Identification of High-Risk Animals

Approximately 30% of long-term infected herds (i.e., more than 1 year as a test-positive herd) have at least one carrier animal that it might be worth trying to identify and cull, because the carrier excretes salmonella bacteria either frequently (active carrier) or more rarely/intermittently (latent carrier) (105, 106). However, it is not easy to correctly identify the carriers, and distinguish them from acutely or transiently infected animals. Persistent carriers typically have persistently high antibody levels (≥80 ODC% in the ELISA-test) over a period of more than 4 months. It therefore requires repeated antibody measurements on blood or milk samples to identify them, and in herds with on-going spread of *S*. Dublin bacteria and poor hygiene, it is not possible to distinguish carriers from animals exposed repeatedly to the bacteria from the environment (99, 105, 106). Some try to detect bacteria in faecal samples from suspected carriers by bacteriological culture or PCR testing. However, the sensitivity is known to be low (<30%) due to the intermittent excretion and low concentrations of bacteria excreted in the faeces, so there is a big risk of getting false negative test-results (88, 93, 94).

### Comparative Issues Concerning the Test Strategies

There are multiple differences between the test strategies, and these differences primarily originate from the pathogenesis and thereby the accuracy of the available tests on herd and animal level. BTM testing can accurately identify BVDV infected dairy herds (81) and *S*. Dublin non-infected herds with little misclassification (98). In contrast, identification of MAP infected herds would be very difficult using BTM antibody detection due to the low within-herd prevalences and the chronic nature of the disease (107), and detection using PCR would require that that detection of MAP in BTM is the target condition desired, which is not the case in the Danish paratuberculosis programme. Detection on animal level is usually very accurate for detection of both BVDV transiently infected and PI animals, with the appropriate combination of tests, although some time may have to pass to testing to be applicable, if pregnant cattle or cattle with colostral antibodies are tested. Accurate detection of *S*. Dublin and MAP infected cattle can be very challenging given the chronic nature of MAP infection and the poorly understood carrier state of *S*. Dublin infected cattle. The differences in herd and animal level test accuracies, with BVD tests being quite accurate, *S*. Dublin intermediate accurate, and detection of MAP infected animals and herds more challenging, makes development of test strategies difficult, but yet possible and worth the effort in relation to communication and educational initiatives supporting the programmes. Combination of tests can be useful for accurate BVDV detection, but for *S*. Dublin and MAP detection, repeated testing is often more useful, which also means a much longer time course to build up evidence of infection status. Furthermore, it means that farmers and veterinarians need to learn about predictive values and how to make decisions in the face of uncertainty, and this is a communication challenge in the programmes.

## Resources, Administration and Legislation

### BVDV

The Danish control and eradication programme was commenced, initially on a voluntary basis in 1994 (66, 108). The efforts of the farmers' own organisations including resources for the organisation and communication of general information about the disease was later supported by legislation and the first BVDV specific ministerial order was issued in 1996. The legislation meant that the disease was notifiable, and emphasis was on health certificates for animals before their movement to other herds or common pastures. Furthermore, focus was on keeping PI animals from pastures, and a systematic test and elimination strategy. Lastly, owners of infected herds should inform neighbours and visitors about their infection status. Over the next years, an additional number of BVDV ministerial orders were issued adjusting different elements of the programme. For example, in 2006, when the eradication programme was changed to a surveillance programme, the initial demands for individual certificates before movement were later replaced by declaration of herd status.

The industry has taken care of the preparation of the risk assessment and management plans for infected herds. If the farmer follows the plan, the industry will pay the costs for blood sampling, lab testing and compensation for euthanized PI animals. This is believed to reduce the eradication period in infected herds and reduce the further risk of spread of BVDV.

### Paratuberculosis

The idea of the Danish paratuberculosis programme was fostered in the Danish cattle sector, who funded and organised research to demonstrate the relevance of the programme and gain experiences with the diagnostic tests and assess risk factors. The Danish Dairy Board (Aarhus C, Denmark) and later on, the Danish Cattle Federation, and subsequently SEGES (the Danish Farmers' central advisory services, Aarhus N, Denmark) organised the programme, which is on a daily basis administrated by the Danish Recording and Milk Yield organisation, RYK (Aarhus N, Denmark) (95). The programme, which was implemented in 2006, was developed as a voluntary control programme aiming to reduce the prevalence of MAP infections in dairy farms in the country and to provide farmers with tools to do so (95). Additionally, a co-operative dairy including 50–100 producers (variable over time) collectively paid the test-costs of all producers following the official programme.

The programme was designed as a test-manage-and-cull programme, where all lactating cattle in all participating herds were tested four times per year. Following testing, the animals are grouped into high-risk and low-risk animals, with further division of high-risk animals into those recommended culled and those that could be kept, but would require additional management to avoid transmission to susceptible calves (95). Only testing to detect antibodies in individual cow's milk (milk-ELISA) have been used to classify cows, and confirmatory testing has been done using follow-up testing with milk 3 months later. Milk samples are collected via the Danish milk recording scheme, and samples are sent to one laboratory only. Milk samples from herds and animals that are due for testing are automatically identified at the laboratory, while the milk recording company submit requests automatically via the Danish Cattle Database to the laboratory. The results of the testing are transferred to the Danish Cattle Database, where test-reports are produced. As such, all reporting of test results is uniform and is the diagnostic testing. Importantly, only one laboratory and one diagnostic test is used (109). This is important because several diagnostic tests could cause confusion when the results differ, which is not uncommon for diagnostic tests for MAP (110).

### *Salmonella* Dublin

The Danish *S*. Dublin programme has been running as a control programme since 2007, with mandatory on-farm control efforts written into the legislation since 2013 and strict animal movement restrictions imposed on test-positive farms. The surveillance and eradication programme is governed by the Danish Veterinary and Food Administration. The programme is financed through the Milk and Cattle Levy Boards, and the daily administration of the programme is performed by veterinarians at SEGES-Cattle, who are in close contact with practising veterinarians and farmers about on-farm control efforts. SEGES also runs projects and advisory services to promote control efforts in the field. Some of these temporary initiatives are free for the farmers, but generally the farmers have paid for local veterinary advice, laboratory testing and control measures themselves. There is close collaboration and dialogue between the veterinary authorities, the cattle sector, laboratories and universities, and the programme and frequent updates of the programme are heavily based on research and data-driven evidence for decision-making in the working group and steering committee.

However, the advice of the researchers and experts is not always possible to follow for political, economic or practical reasons. Currently, the decision has been to increase the pressure to control the infection by letting the veterinary authorities visit test-positive farms that do not manage to improve their status. The authorities can under given conditions give the farmer injunctions to seek special veterinary advice about how to better control the infection from second opinion veterinarians approved by the Veterinary and Food Administration to consult on *S*. Dublin control measures.

### Comparative Aspects of Resources, Administration and Legislation

For all diseases, the availability of one laboratory running most of the analyses allowing for clearer interpretation (with a known, but not perfect level of test-accuracy) is deemed to have limited the confusion about test-results that might otherwise differ between laboratories and cause frustration among users. There has been considerable differences in how tight the programmes have been followed up by legislation. For BVDV, legislation was introduced already 2 years after the voluntary programme was initiated. For *S*. Dublin legislation was introduced from the beginning of the surveillance period in late 2002, and it was tightened several times between 2008 and today.

European management strategies for non-EU-regulated diseases that mainly have economic consequences for the farmers, have developed in a direction that places more responsibility for disease prevention on the individual farmers. This may leave the initiatives less organised and coordinated, which again might lead to lack of the required long-term and focused engagement.

## Feasibility in Practise

### BVDV

The key transmission routes were known, when the Danish BVDV programme was initiated. However, there was a need to demonstrate that control and eradication could be carried out in practise. This was demonstrated in a so-called “island-project” including all 36 dairy and 77 non-dairy herds in the island Samsø, where all farmers agreed to participate (91). It was demonstrated that eradication was possible if the risks due to trade of cattle and contact transmission on neighbouring pastures were addressed. This implied avoiding contact with PI animals, isolation of purchased animals, no pasturing if there were PI animals on neighbour fields, control of common pastures, animal exhibitions and livestock markets, and finally farmer compliance to follow guidelines was very important. Vaccination against BVDV has never been used in Denmark and was specifically forbidden according to legislation in 1996, i.e., the Danish legislative order BEK 1279 19/12/1996. Furthermore, the Danish Dairy Board had their own laboratory, which could readily run the all the analyses following years of experience with the programme on IBR, which was established in the 1980'ies. The majority of testing could be done at this laboratory using only one set of tests and one set of interpretations. This laboratory supplemented the national reference laboratory, which also checked the accuracy of the test-results.

### Paratuberculosis

There are three main challenges in the Danish programme on paratuberculosis: (1) the programme is voluntary, and not all farms are included, which probably is due to lack of motivation; (2) the frequent testing may be an obstacle from a cost-point of view and also from a logistics-point of view. However, because milk samples from the milk recording system are used, the logistic challenges have been few, because “all” the farmer had to do was sign up for the programme and use the interpreted test-results, which were presented ready for management (83). The laboratory capacity was relatively quickly sufficient to handle the 500,000–800,000 samples per year that were received after the first year in the programme. Test costs is mostly an issue of a benefit-cost analysis, and it is not clearly logical for the farmers to be in the programme if there is no documented high prevalence in the herd. In 2020, a surveillance component was added to the programme to reduce the test costs for farmers with a low within-herd prevalence; (3) adhering to the risk mitigating actions required to break transmission routes. This key area is a challenge and will be discussed further.

The key recommendations for risk-mitigation are to: (1) cull all cattle that are repeatedly test-positive in milk ELISA prior to next calving; (2) if these cows and any other cows with single test-positive results are used, measures should be taken to reduce transmission of MAP via faeces i.e., primarily in the calving pen; (3) use of milk and colostrum should be done from repeatedly test-negative animals only; and (4) purchase of livestock should be avoided, but if done anyway, it should be from herds with known low prevalence of test-positive animals. Culling of repeatedly test-positive cattle will remove cattle at high risk of shedding MAP. Nonetheless, far from all cattle are currently culled prior to next calving, despite that removal of the key source of the pathogen transmission would be the desired result. If these animals are kept despite this risk, it is important to reduce contact time to their manure, and to let these cows calve in separate calving areas. Whereas, half of farmers have the cows calve in separate calving areas, cleaning of the calving pen is done by less than half of the farmers. Furthermore, removal of the calf is not done as frequently as recommended. The lack of compliance may be because these important risk mitigation measures take up too much of the farmers' time, and are difficult to implement. In contrast, the avoidance of use of milk and colostrum from test-positive animals is easy to implement and therefore done by the majority of farmers in the programme (111). This illustrates that the feasibility with which farmers can implement suggested risk mitigation measures is really important.

### *Salmonella* Dublin

The prevalence of test-positive dairy herds has been hovering around 8–10% for several years, and new infections and outbreaks of disease in naïve herds as well as reinfections or resurgence of infections are still evident. There are clear clusters of infection transmission on-going in cattle dense geographical areas of the country, and it is likely that the diffuse environmental spread through manure and vehicles of local and regional spread of contaminated manure are creating challenges that are difficult to clearly identify, and to track and trace in the current control programme.

Another challenge is a lack of incentives for some of the infected farms. Controlling *S*. Dublin requires focused, long-term and daily manual work to keep the environment sufficiently clean and to house the animals in ways that prevent spread of the bacteria (88). This may be costly in some farms that need to implement changes in the management and/or housing facilities. At the same time, clinical signs are far from always clearly associated with *S*. Dublin in the rapidly expanding and growing Danish cattle farms, where many other infectious diseases also cause diarrhoea, respiratory disease and ill-thrift in calves, as well as abortions in adult cows.

Production losses may not be visible to the farmer, as they may not affect single animals dramatically unless a cow gets clinical salmonellosis. Rather the losses are typically expressed as a general reduction in the milk yield over time that prevents the cows from reaching their full genetic potential compared to non-infected farms. This can be caused by the infection in cows, or as a delayed effect of respiratory disease in the calves that lead to reduced milk-yield in the first lactation (112). It has been estimated that economic penalties (or benefits) that would differentiate the milk and beef prices paid by the dairy and meat plants by 1% increasing to 5% over a few years would have an expected marked effect on the incentives to control *S*. Dublin in the infected farms (113). One of the small dairy companies do pay 1% lower price for the milk delivered from test-positive herds showing that it is possible to implement. However, the approach has so far been rejected by the big companies due to practicality issues and concerns about international competitiveness. Furthermore, it is not an easy decision to take to implement economic incentives during times where the milk and beef prices are low, and the cattle sector is under different types of societal and market pressures.

The test scheme used in the surveillance programme and for control efforts may also pose a challenge, as some infected farms may go undetected for too long if the BTM test is not able to detect few infected cows in a large herd, or if infection starts among young stock, which are not tested. Thereby false-negative herds may be spreading the infection, whether it is unknowingly or not. The programme is likely to be changed during 2021 to improve the classification scheme and provide improved protection of the non-infected cattle farms.

### Comparative Aspects of Feasibility

The feasibility differs significantly between programmes, but the exact differences may be difficult to appraise without having insight into the national/sectorial decision making processes. Here, the three diseases may have been relatively similar: the agricultural organisations have set out to determine the relevance and feasibility, they have taken the decision and then moved on. One of the tools in this decision process has been use of pilot projects. A specific pilot project proving the effectiveness of a control programme in a geographical defined area was carried out for BVDV, where the disease could be eradicated from a defined island cattle population relatively easily. For *S*. Dublin, a regional pilot project in a Southern Jutland high cattle density area demonstrated in 2007–2008 that such a centrally organised project could promote voluntary control activities in participating dairy herds with stable school-like networks of farmers focusing their efforts on risk mitigation, biosecurity and herd specific test strategies (114). Although the results were encouraging, this pilot project did not have the same clear effect as the BVDV pilot project. The *S*. Dublin pilot project was not followed for long enough to be able to evaluate the effect in all the participating farms. A similar approach would have been difficult for MAP, because of the more protracted course of infection, and lack of ability of including all farmers in a region for a sufficient long time. Notably, this would have lasted more than a cattle generation (5–10 years or more) for paratuberculosis, whereas it could be achieved in a few years for BVDV. Therefore, for paratuberculosis and *S*. Dublin, feasibility was assessed via voluntary herds, where the proof of concept was demonstrated (77).

Pasture control was strict in island-pilot project for BVDV, but is less controlled in *S*. Dublin programme today, a point that may appear illogical. However, the changed cattle population structure and new needs for outdoor housing and mandatory pasturing of organic farms complicate very strict regulation on pasturing of animals from infected herds. Double fencing is recommended and discussions are ongoing about how it might be made mandatory by legislation in the *S*. Dublin programme working group. However, it is not trivial to keep heifers and cows behind fences in all areas of the country, nor to control fencing regulations.

A major difference may be the time, when the programmes were established. From 1994 to 2020, the number of cattle decreased from 2.2 to 1.5 M heads. When the BVDV programme was established in 1994, there were around 660,000 dairy cows in approximately 16,000 herds (average herd size: 42 cows), while in 2019, there were 563,000 dairy cows in 2,800 dairy herds (average herd size: 200 cows). Thus, larger units prevail making control of infectious diseases a challenge (86). Furthermore, the motivations for controlling the diseases also differed markedly. While production economy was a key driver for BVDV, the zoonotic aspect and animal welfare issues were initially the main drivers for *S*. Dublin, later added production losses as an increasingly more important driver. The motivations for control of MAP may be somewhat in between.

## Discussion

We have described purposes, principles, design and tools used in the programmes on BVDV, *S*. Dublin and MAP in Denmark. As summarised in Table 2, BVD has been successfully eradicated, whereas the decline in the prevalence of *S*. Dublin has halted (Figure 3) and the decline in the prevalence of infected MAP herds (Figure 2) has plateaued and only reached participating herds, of which there are fewer. The most likely reason for this difference is that BVD is an acute viral disease with clear routes of transmission that are easily broken with an effort effectively working within a few years only, if appropriate measures are taken. Most farmers can stay motivated for that time-period. Control can take longer, e.g., a cattle generation (6–8 years) for MAP and *S*. Dublin, which both spread and survive in the environment. Not all farmers and veterinarians can keep up motivation and focus on the control measures for that long. Furthermore, lack of accurate diagnostic tests makes monitoring of progress a challenge. Still, the use of inexpensive tests can provide some information, that can be useful to monitor the progress with some uncertainty. Acceptance of uncertainty in test-interpretation among farmers and veterinarians is a prerequisite in the control of these types of infections.

The three programmes have many similarities and many differences (Table 2). Firstly, the similarities are based on the organisation of the programmes as run by the farmers' organisations and using tools, instruments and communication primarily done via these organisations in collaboration with researchers from the universities in charge of education of veterinarians and veterinary preparedness for the authorities and the Danish Veterinary Association. The farmers' organisation has partly defined the objectives of the programmes. However, here the differences also start to be evident, as *S*. Dublin is a zoonosis. Therefore, the veterinary authorities have a key interest in the objectives and the chosen approach to control the disease, and the human health institute, SSI, is frequently consulted to follow the development in human cases closely. Still, the progress of the programmes are to a large extent driven by the farmers' organisation, where the leaders are well aware that Denmark is a food producing and exporting country, and that high-quality products are essential for the continued export and for opening new markets.

Other differences between the programmes are rooted in the differences of the pathogens and associated pathogeneses and environmental survival (Table 1). BVDV readily spread, but also cannot survive for long outside the hosts. Therefore, risk mitigation measures are important to control the infected animals, which are easily identifiable. Both MAP and *S*. Dublin can survive for extended periods outside the hosts, and measures to address this is important. They are not as infectious, and all bacteria may not have to be eliminated from a herd for the infections to eventually die out. However, diagnostic tests with accuracies that are far from perfect make identification of pathogen reservoirs a challenge, and therefore continued spread is likely to occur, if continued surveillance for early detection is not carried out. This also means that farmers need to be motivated for longer periods of time, often more than a cattle generation (5–10 years) can be needed. Motivation is therefore a key factor that cannot be ignored. Motivational initiatives such as financial incentives that can be expected to be effective may not always be desirable to implement for other reasons (e.g., market drivers, economic or organisational constraints) and they might require that several stakeholder agree to implement incentives simultaneously. For BVDV, it appeared that a strong motivation was build up over a relatively short period. Hence, it could be hypothesised that focus on one clear driver (e.g., production economy) is easier to communicate to farmers and in that sense can be more efficient.

While the reasons for participation may differ between the programmes due to the voluntary or mandatory nature of the programmes, this is also the case when looking at international literature. There is a lot of focus and programme activities to combat BVDV throughout the world. There is also increasing spread of *S*. Dublin—even multi-drug resistant types—and therefore concerns and increasing focus on how to control *S*. Dublin in many different countries (99). For MAP control, most countries are generally focusing on animal health, then reduction in production losses, followed by maintenance of trade, animal welfare and lastly public health (23). However, key reasons for participation also lie in the motivation for controlling the diseases. Financial impact of the diseases are obviously important, because farmers are easier to motivate if they can see an immediate financial benefit. However, many farmers also care about their animals, their health and welfare, and they know they are food producers (54). Consequently, these aspects weigh in as well.

It can be argued whether “control” or “eradication” is the most viable approach, and control can for some diseases be as ideal as eradication (115). However, for highly infectious diseases such as BVDV, it can be difficult to contain the virus, and eradication may be a more obvious choice. Vaccination could be an alternative. However, there has never been a strong drive for use of vaccines in the cattle sector when eradicating diseases in Denmark. For BVDV this option was explored, but due to a failure in demonstrating effectiveness (66), an approach without use of vaccines was taken, irrespective that others have subsequently found use of vaccines for BVDV control (116). No vaccines are available to effectively control the spread of *S*. Dublin (99), and the vaccines for MAP were banned in Denmark in 2008 due to their interference with *Mycobacterium bovis* testing. Still, use of vaccines can be a strategic choice for some diseases, as are many of the other choices made in disease control. The background for these choices are at times clear, but at other times the result of political negotiations and events that are not really obvious from a scientific point of view, or they may not have been elucidated. Such processes have to our knowledge not been described in the scientific literature.

## Author Contributions

All authors contributed to the design of the study and performed literature reviews, as well as the writing and approval of the final manuscript.

## Conflict of Interest

The authors declare that the research was conducted in the absence of any commercial or financial relationships that could be construed as a potential conflict of interest.
